# Analysis of Adsorbed Polyphosphate Changes on Milled Titanium Dioxide, Using Low-Field Relaxation NMR and Photoelectron Spectroscopy

**DOI:** 10.1021/acs.langmuir.2c03416

**Published:** 2023-04-13

**Authors:** Laura N. Elliott, David Austin, Richard A. Bourne, Ali Hassanpour, John Robb, John L. Edwards, Stephen Sutcliffe, Timothy N. Hunter

**Affiliations:** † School of Chemical and Process Engineering, University of Leeds, Leeds LS2 9JT, U.K.; ‡ School of Chemistry, University of Leeds, Leeds LS2 9JT, U.K.; § Venator Materials PLC, Titanium House, Hanzard Drive, Wynyard Park, Stockton-on-Tees TS22 5FD, U.K.

## Abstract

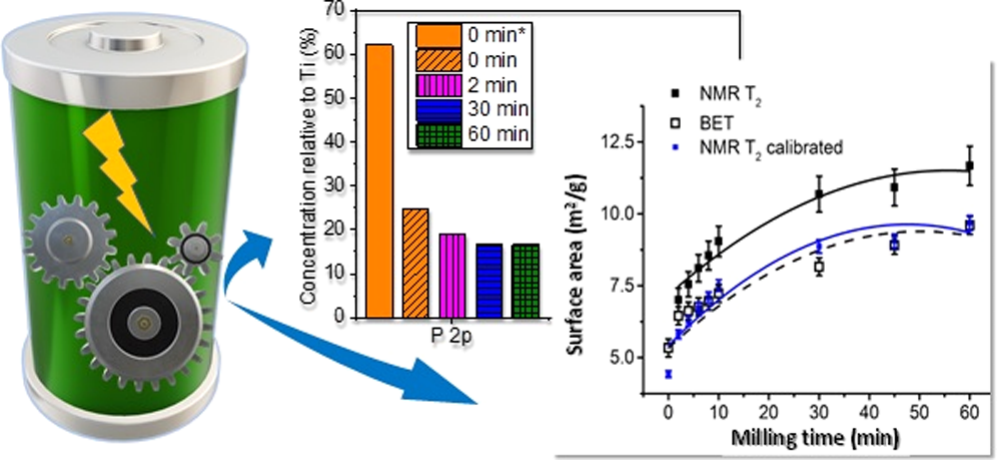

In this study, changes in the adsorbed amount and surface structure of sodium hexametaphosphate (SHMP) were investigated for aluminum-doped TiO_2_ pigment undergoing milling. Relaxation NMR was utilized as a potential at-line technique to monitor the effect of milling on surface area and surface chemistry, while XPS was used primarily to consider the dispersant structure. Results showed that considerable amounts of weakly adsorbed SHMP could be removed with washing, and the level of dispersant removal increased with time, highlighting destructive effects of sustained high-energy milling. Nonetheless, there were no significant chemical changes to the dispersant, although increases to the bridging oxygen (BO) peak full width at half-maximum (FWHM) suggested some chemical degradation was occurring with excess milling. Relaxation NMR revealed a number of important features. Results with unmilled material indicated that dispersant adsorption could be tracked with pseudo-isotherms using the relative enhancement rate (*R*_sp_), where the *R*_sp_ decreased with dispersant coverage, owing to partial blocking of the quadrupolar surface aluminum. Milled samples were also tracked, with very accurate calibrations of surface area possible from either *T*_1_ or *T*_2_ relaxation data for systems without dispersant. Behavior was considerably more complicated with SHMP, as there appeared to be an interplay between the dispersant surface coverage and relaxation enhancement from the surface aluminum. Nevertheless, findings highlight that relaxation NMR could be used as a real-time technique to monitor the extent of milling processes, so long as appropriate industrial calibrations can be achieved.

## 1 Introduction

Rutile phase titanium dioxide (titania) is a key pigment used in a wide range of consumer products, such as paints and cosmetics, owing to its brilliant whiteness and hiding power.^1,2^ Despite its popularity, the manufacture of high-quality titania powder is complex, including several interactions with secondary species. Dopants such as AlCl_3_ are added during the synthesis to enhance rutile formation and reduce agglomeration, leading to aluminum-substituted crystal planes and resultant surface chemistry changes.^3,4^ Dispersants, often polyphosphates, are also added to reduce secondary aggregation prior to wet milling, as essential steps to maintain critical precise particle sizes (typically ±5 nm) in the range of ∼200 to 300 nm for optimal light reflection.^1,5−7^

The use of dispersants with TiO_2_ during the milling process leads to several processing complications. For example, it is relatively unknown as to what extent milling time and temperature may modify dispersant coverage or drive degradation reactions (e.g., partial hydrolysis) once adsorbed onto the TiO_2_ surface. Also, with the high energy cost of milling, it is imperative a more complete understanding is gained into both chemical and physical changes that occur over time to ensure sustainability goals are achieved. Therefore, the use of an inline or at-line technique may provide invaluable information for industrial optimization and quality control.

The use of polyphosphates for mineral processing is widespread.^8−10^ Notwithstanding this, it is also known that aqueous polyphosphate solutions are not stable under several process conditions, such as high temperature, acidic or basic environments, and in the presence of certain metal ions.^8,11,12^ Smaller phosphates, for example, orthophosphates, can be formed by hydrolysis that will have an impact on the dispersion properties of the system.^8^ Importantly, the polyphosphate chain length has an impact on steric stabilization, with evidence suggesting that polyphosphates with unit number *n* > 4 have better dispersion properties than those of shorter chain length.^13,14^ This reduction is because steric stabilization requires a proportion of the polyphosphate chain to extend into the solvent, resulting in increased particle–particle repulsions.

Industrial milling can additionally cause an increase in temperature to at least a few tens of degrees, which has also been known to cause hydrolysis.^15^ Thus, it may be that overmilling itself may cause critical dispersant degradation. Despite this potential issue, the rate of hydrolysis and its impact on the dispersion properties of pigment has not extensively been investigated. However, the current authors have previously found evidence that industrially milled titania dispersed with sodium hexametaphosphate significantly changed with milling time, where substantial removal of the polyphosphate was evident.^3^ It was theorized that some degree of removal may have been from partial hydrolysis, although the complex conditions of the industrial process made it difficult to confirm any chemical changes.

X-ray photoelectron spectroscopy (XPS) has previously been demonstrated to be a key technique in understanding the adsorption behavior of polyphosphates on mineral surfaces^12,16,17^ and thus potentially to study the effects of milling. Zhang et al.^16^ determined the P-to-Al atomic ratios before and after treating kaolinite and diaspore (an aluminum oxide hydroxide mineral) with acid. The acid-treated kaolinite led to a reduced adsorption capacity, while the treated diaspore adsorbed more polyphosphate. The authors related these differences to changes in the number of Al sites, where an increase in unsaturated Al was observed after acid treatment of diaspore, thus allowing enhanced polyphosphate interaction. Ni and Liu^17^ also used XPS to analyze the adsorption behavior of the same polyphosphate on pyrochlore and calcite, where binding energies of Ca and P indicated that the hexametaphosphate groups formed a complex with calcium on calcite, but no chemisorption was measured with pyrochlore. More recently, Kasomo et al.^18^ used XPS to study polyphosphate adsorption onto pure rutile TiO_2_, as well as almandine, where low chemical shifts inferred relatively weak chemisorption of the dispersant onto rutile surfaces. It is unknown whether milling may alter the balance of chemisorption versus physisorption.

Nuclear magnetic resonance (NMR) relaxometry is also proving to be an invaluable technique to study dispersion surface properties,^19^ or otherwise applied industrially to analyze pore structures and wettability in reservoir rocks for enhanced oil recovery,^20−22^ where the use of very low-field, desktop instruments has provided an avenue for inline or at-line analysis. Originally, the technique was largely developed for rapid surface area measurements of nanoparticle dispersions^23,24^ but has advanced extensively to incorporate analyses of a number of colloidal surface chemistry effects^25−27^ and even nanobubbles.^28^ For example, recent studies have utilized the technique to evaluate the exfoliation or surface chemistry of graphene oxide and other related carbon materials^29−31^ as well as the stability and aggregation of various mineral dispersions,^32−34^ and influence of ultrasonication on dispersion,^35^ where it has also pointedly been used to quantify organic and inorganic dispersants.^36−39^ It has also been used to characterize different TiO_2_ nanocrystal polymorphs.^40^ Additionally, contemporary work by Shakirbay et al.^41^ considered the use of relaxation NMR to characterize milled alumina slurries, showing *T*_2_ relation rates correlated with the level of mixture dispersion from other particle characterization methods. Indeed, previous work by the current authors explored the suitability of the technique to track changes in industrially milled titania dispersions.^3^ Here, the relaxation rate of washed milled samples was greater than unwashed samples, which was assumed to be due to differences in milled dispersant density. More generally, the technique offers the potential for rapid *in situ* analysis of mechanochemistry reactions and crystallographic phase changes produced in ball milling, as an alternative to current Raman and X-ray techniques.^42^

Therefore, to increase our understanding of dispersant interactions within these complex industrial systems, here, polyphosphate changes in titania pigment suspensions were investigated for systems undergoing wet media milling. Importantly, presynthesized aluminum-doped titania dispersions were utilized in a bespoke laboratory mill, where conditions were able to be closely controlled, and systems with and without sodium hexametaphosphate dispersant could be studied. A critical aim was to establish whether excess milling energy caused any physical removal of the dispersant or partial chemical degradation. For this, XPS was used to measure the changes to phosphorus and aluminum content as milling progressed, as well as their chemical environments. Additionally, NMR relaxometry was used to examine the influence of both surface area changes and polyphosphate–alumina interactions on relaxation times. A key objective was to consider how such a technique could potentially be used as an at-line process control tool for the online monitoring of TiO_2_ particle size during milling.

## 2 Materials and Methods

### 2.1 Materials

Nonmilled rutile TiO_2_ (titania) reactor discharge, containing aluminum burned into the crystal, was supplied by Venator Materials PLC as a slurry. It is an industrial-grade pigment that was obtained prior to site polyphosphate dosing and was purified by washing three times in deionized water (5 L aliquots) to remove impurities. The suspensions were left to sediment by gravity over 2 weeks before removing the supernatant, whereupon the TiO_2_ solids were dried in an oven at 100 °C overnight. Suspensions were then made as required from the dried powder, using deionized Milli-Q water (Merk-Millipore).

Sodium hexametaphosphate (SHMP, as abbreviated throughout) was used to control the TiO_2_ dispersion properties (purchased from Sigma-Aldrich, U.K.). SHMP has the general chemical formula (NaPO_3_)*_n_*, where the average chain length *n* = 6. Also, 1 M HCl and 1 M NaOH stock solutions were used to adjust the pH of the suspensions, while NaCl was used as a background electrolyte (all Fischer Scientific Ltd, U.K.).

### 2.2 Polyphosphate Adsorption on Unmilled TiO_2_

100 mL of various known initial concentrations of SHMP were prepared at pH 4 in background electrolyte (1 mM NaCl). Then, 0.1 g of the unmilled titania was added and mixed for over 1 h, with the pH adjusted if required to maintain pH 4 conditions. Initially, the pH was observed to drift slightly toward more alkaline conditions, although, values generally stabilized after 10–15 min. The samples were then left on a roller shaker (IKA, U.K.) for 12 h, after which the pH was checked once again, where it is believed that 12 h is far in excess of the time required for equilibrium adsorption to occur. Previous work by Taylor et al.^43^ on similar doped TiO_2_ and SHMP systems, indicated equilibrium was reached in tens of minutes, which would be consistent with other electrostatic-driven adsorption of large molecules on mineral systems.^44^

After adsorption, samples were centrifuged on a Heraeus Megafuge 16R (Thermo Scientific) at 10,000 rpm for 30 min, whereupon the supernatant was extracted and passed through a 0.22 μm syringe filter to remove any particles that remained. The supernatant was analyzed via ICP-OES (iCAP7600, Thermo Scientific, U.K.) with a five-point calibration made using SHMP solutions at pH 4 and 10 mM NaCl. Adsorption was quantified by fitting to a Langmuir monolayer isotherm^45,46^ with full details within the Supporting Information (SI). All adsorption experiments were conducted in a temperature-controlled environment at 21 ± 0.5 °C.

Samples also were prepared using this method to obtain analogous pseudo-isotherms by relaxation NMR (see Section 2.6 for technique details) with the exception that the dispersions were not centrifuged, and the supernatant was not removed. Instead, aliquots (containing particles, adsorbed and unbound SHMP) were removed after the samples were left on the shakers overnight and were measured using the method also outlined in Section 2.6.

### 2.3 Preparation and Milling of Polyphosphate-Adsorbed Titania

To prepare dispersions for milling and characterization, 55 g of 1 mM NaCl, pH 4 stock solution was added to 20 g of the dried TiO_2_ powder and mixed for 30 min with a magnetic stirrer and plate. A solution of SHMP was prepared by adding 1.4 g of SHMP to 15 g of 1 mM NaCl, pH 4 solution. The addition of SHMP caused the pH of the background electrolyte to increase, so HCl was added dropwise to decrease the pH back to 4. The solution was then vigorously shaken to fully dissolve the SHMP and added to the TiO_2_ dispersions. The SHMP was left to adsorb onto TiO_2_ for 1 h with continuous stirring, and the pH was monitored and again adjusted back to pH 4 if required. Thus, the final TiO_2_ slurry was 22 wt % and the total initial concentration of SHMP was at 20,000 ppm, which is in excess of the maximum adsorption estimated from previous research.^5^

The TiO_2_-SHMP slurries were then milled in a stainless-steel grinding chamber with a ceramic spindle, which was fabricated to fit an L5M-A high shear mixer (Silverson Machines Inc). Milling beads (zirconia-coated silica, 500 μm) were first added to the chamber, to give a fill ratio of 0.8. Initially, the mill was set to 100 rpm for 1 min to fully coat the milling beads with the TiO_2_ slurry. The rpm was then adjusted to 6000 rpm (which has previously been found to give optimal milling performance in similar systems^5,45,46^) and the slurries were milled for various residence times (*t* = 2–60 min). After milling, the slurries were removed from the chamber and the grinding media was washed in deionized water and sieved between 350 and 560 μm mesh sizes, to remove any potential fragmented milling beads prior to reuse. Titania-only slurries (without any addition of SHMP) were also milled using the same methodology for comparison.

The milled slurries were then prepared and dried for characterization, using XPS, NMR, and other methods detailed. First, they were washed and separated via centrifuge in three cycles to remove any nonadsorbed SHMP. After the final wash cycle, the supernatant was removed, and the remaining suspensions were left to dry in a glass drying oven held at 50 °C for approximately one week. A nonmilled TiO_2_ sample with SHMP adsorbed was also left unwashed and dried, in order to investigate if the washing procedure effected the SHMP structure, although, any nonadsorbed SHMP was then dried onto the particle surface.

### 2.4 Particle Characterization

A Zetasizer Nano ZS (Malvern Panalytical, U.K.) was used to measure the ζ potential of the milled and dried TiO_2_-SHMP dispersions, by preparing at 100 ppm in Milli-Q water with 0.1 mM NaCl. A separate sample from a freshly prepared dispersion of TiO_2_ and 20,000 ppm SHMP was also prepared to examine whether the drying procedure affected the dispersion properties at all. Also, the ζ potential was measured for an unmilled dispersion that was heated at 100 °C for 1 h, to establish whether the SHMP may degrade from extended heating. Stock base (NaOH) and acid (HCl) solutions were prepared at 0.1 M, to increase and decrease the pH of the particle dispersions, respectively. Further characterization of unmilled dispersions prepared the same way was performed using an analytical centrifuge (LUMiSizer, LUM GmbH) where the linear settling rate was tracked for samples under 300 rpm.

For samples without SHMP, Brunauer–Emmett–Teller (BET) surface area measurements were obtained by nitrogen adsorption-desorption at 77.3 K, using a TriStar 3000 (Micromeritics) surface analyzer. Milled and dried titania samples were initially degassed to remove moisture using a vacuum oven at 110 °C for a minimum of 12 h and a maximum of 18 h (no observed changes in mass occurred when leaving for longer periods) under a vacuum of 10 mmHg. Particle size distributions were also measured using a Mastersizer 2000E (Malvern Panalytical, U.K.) using a Hydro2000SM aqueous dispersion cell (for sample volumes between 50 and 120 mL). Here, milled and dried TiO_2_ powder was initially made into 15 wt % dispersions, which were then subsampled into the measurement chamber to the correct obscuration. Measurements were completed for dispersions with and without adsorbed SHMP.

### 2.5 X-ray Photoelectron Spectroscopy (XPS)

An Axis Ultra DLD system (Kratos Analytical Ltd.) was used to collect XPS spectra using a monochromatic Al Kα X-ray source operating at 150 W (10 mA × 15 kV). Data from the dried milled samples were collected with pass energies of 160 eV for survey spectra, and 40 eV for the high-resolution scans with step sizes of 1 and 0.1 eV, respectively. The system was operated in hybrid mode, using a combination of magnetic immersion and electrostatic lenses, and acquired over an area of approximately 300 × 700 μm^2^. A magnetically confined charge compensation system was used to minimize charging of the sample surface, and all spectra were taken with a 90° take-off angle. A base pressure of ca. 1 × 10^–9^ Torr was maintained during the collection of the spectra.

Data were analyzed using CasaXPS v2.3.21 (Case Software Ltd.) after subtraction of a Shirley background and using modified Wagner sensitivity factors as supplied by the manufacturer. Note that due to the overlap of the Na(1s) core level with a portion of the Ti Auger signal, a model for this background signal was obtained from TiO_2_ and fitted in addition to a Na(1s) component.

### 2.6 NMR Relaxometry

Low-field NMR relaxation studies were performed on an Acorn Area 13 MHz desktop spectrometer (Xigo Nanotools) as detailed in previous investigations.^3,19,26^ Both spin–lattice (*T*_1_) and spin–spin (*T*_2_) relaxation time measurements were made to understand how the device could be used to monitor dispersion surface area measurements as well as the influence of SHMP on relaxation enhancement.

For all relaxation measurements, a 90° pulse length of 5.67 μs and a 180° pulse length of 11.33 μs were used with a gain value of 10 dB (as per protocols used previously for similar systems^3^). A *T*_1_ inversion recovery pulse sequence was used to measure the spin–lattice relaxation rate coefficient, with a recycle delay of 5*T*_1_ between scans. An iterative process was initially used to determine an anticipated *T*_1_ to within 20% of the ‘true value’. A Carr–Purcell–Mei boom–Gill (CPMG) pulse sequence was used to measure the spin–spin *T*_2_ relaxation, where typically, a series of four replicate scans were averaged to produce the CPMG trace. The Xigo Nanotools software fitted the collected signal to a single exponential to extract the *T*_1_ or *T*_2_ relaxation time. As for the adsorption studies, all relaxation measurements were performed at 21 ± 0.5 °C

The method considers an initial calibration to define a specific calibration constant, *K*_a_ (g/(m^2^·ms)), as a function of the specific measured relaxation rate ratio, *R*_sp_ (unitless), specific surface area, *S* (m^2^/g), particle volume fraction, *Φ*_*p*_, and relaxation rate of the bulk solvent, *R*_b_ (ms^–1^). We note *R*_b_ = 1/*T*_1,2_ (where *T*_1,2_ is the measured spin–lattice or spin–spin relaxation time). If *K*_a_ is defined from an initial calibration system of known parameters, then the equation can be rearranged to determine changes in surface area (*S*).
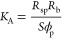
1The specific relaxation rate ratio, *R*_sp_, is calculated as per eq 2. Here, *R*_av_ (ms^–1^) is the measured relaxation rate, while *R*_*i*_ is the measured relaxation rate of initial conditions. This is normally of the bulk solution (*R*_*i*_*= R*_b_), although more qualitatively, the relaxation rate of the initial dispersion may also be used to internally track changes within a system.
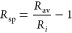
2For unmilled TiO_2_-SHMP samples, dispersions were prepared, as per Section 2.2. The measured relaxation rate (*R*_av_) of the initial dispersion (without SHMP adsorbed) was used as *R*_*i*_, and the *R*_sp_ changes were tracked through measurement of the relaxation rate with increasing concentration of SHMP.

For milled TiO_2_-SHMP samples, suspensions were prepared at 15 wt % from the washed and dried powders (Section 2.3) at pH 4, 7, or 9, adjusted dropwise with NaOH. Prior to analysis, samples were dispersed in an ultrasonic bath (Clifton Sonic) for 30 min, followed by 2 min on a Model 505 Sonic Dismembrator ultrasonic probe (Fischer Scientific) at 30% amplitude. Samples of TiO_2_ that were milled in the absence of SHMP were also evaluated to study the technique’s ability to gain at-line surface area measurements. Here, the calibration constant, *K*_a_, was calculated using the BET surface area of the unmilled (0 min) TiO_2_ sample and measured *R*_sp_, using a separate measurement of the bulk solvent relaxation rate, *R*_b_. The *K*_a_ was then assumed to be constant with milling time, as it was expected to be only affected by surface chemistry changes rather than surface area.

## 3 Results and Discussion

### 3.1 Adsorption of Polyphosphate onto Unmilled Titania

ζ Potential values for the unmilled Al-doped TiO_2_ are shown within the SI (Figure S1), where the isoelectric point (i.e.p.) was measured at ∼pH 6 and in good agreement with the literature.^47^ The surface charge of the TiO_2_ at pH 4 is approximately +30 mV while at pH 9 it is approximately −45 mV, which are also consistent with previously reported literature,^4^ although are slightly shifted, likely due to the particles having a higher Al surface coverage than the samples used herein.

SHMP is highly negatively charged, and thus when the TiO_2_ is at pH 4, it is likely that electrostatic adsorption will dominate between the negative phosphate groups and positive AlOH^2+^ and TiOH^2+^ sites at the particle surface. Conversely, when the TiO_2_ is at pH 9, the electrostatic interaction is repulsive. Despite this, the literature has shown that polyphosphate adsorption still occurs^5,14,43^ (with recent evidence showing only an ∼30% reduction for very similar systems^5^) indicating that adsorption in these conditions occurs via a chemisorption mechanism. At pH 9, the Al-doped TiO_2_ surface will comprise neutral AlOH groups, positive AlOH^2+^, and negative TiO^–^. In this case, electrostatic interactions between the particle and polyphosphate may occur with the AlOH^2+^ sites, but these are not favorable with the negative titanium hydroxide surface sites that dominate the surface, resulting in the overall electronegativity. Nevertheless, chemisorption may occur through bidentate surface complexation leading to the formation of Al–O–P and Ti–O–P bonds.^43^

**Figure 1:**
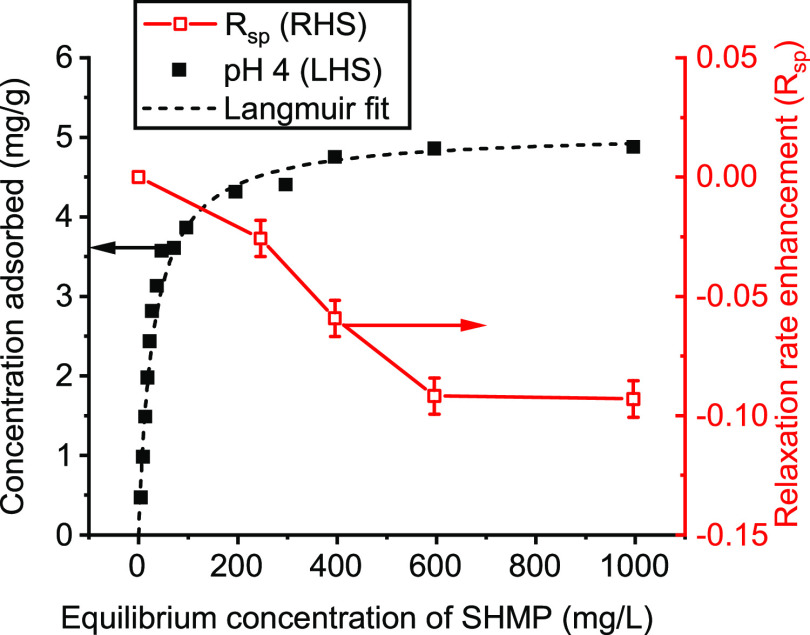
Adsorption of SHMP on unmilled titania by ICP-OES (black, closed) where the black dashed line is the Langmuir isotherm fit. Pseudo-isotherm (red, open) generated from the NMR relaxation rate enhancement (*R*_sp_) calculated using the *T*_2_ relaxation time (*R*_sp_ = *R*_2sp_). *R*_sp_ is scaled to an equivalent sample of the bare particle to remove the effect of the particle surface area. The connecting line (red) is a guide to the eye.

The adsorption isotherm of SHMP adsorbed onto Al-doped TiO_2_ at pH 4 is shown in Figure 1, which includes a monolayer fit using the Langmuir model^46,48^ (see the SI, Figure S2). It can be observed that the adsorbed SHMP plateaus at equilibrium concentrations of ∼600 ppm with a fitted maximum adsorption of 5.03 mg/g (SI, Table S1). Relaxation NMR is a technique that has previously been used to generate pseudo-isotherms for various systems^25,38,49^ and so Figure 1 also presents the NMR pseudo-isotherm by plotting the change in qualitative *R*_sp_ from *T*_2_ relaxation times (*R*_2sp_, using the initial dispersion for *R*_*i*_, rather than that of the bulk solvent).

Regarding the adsorption data, Table S2 (SI) compares maximum adsorbed amounts to that found by Taylor et al.^43^ in similar work. Comparing adsorption density to the specific surface area of the unmilled particles (as determined using BET) a value of 0.95 mg/m^2^ was calculated in this study, which is almost double that found previously. The likely reason for the difference in adsorption behavior is due to the difference in polyphosphate chain length. Here, SHMP has an average length *n* = 6, compared to that used by Taylor et al.^43^ who used a polyphosphate with a chain length *n* = 10–12. It is reasonable that the maximum adsorbed amount would be lower for a doubling in the chain length from increased molecular hindrance, and this is also in agreement with other work of Michelmore et al.,^14^ who explored polyphosphate adsorption onto TiO_2_ with varying polyphosphate chain length.

Interestingly, with respect to the NMR relaxation data, the *R*_2sp_ is reduced as SHMP adsorption increases toward the plateau value. The addition of a dispersant or polymer to a particle surface would normally be expected to increase the relaxation rate because of the reduction in the rotational motion of surface-bound water.^25^ However, Cheesman et al.^50^ previously showed a decrease in the *R*_sp_ for an increase in a macroinitiator adsorbed on silica nanoparticles, due to the displacement of water from the silica surface upon adsorption of the macroinitiator. Reductions in *R*_sp_ have also been reported with the addition of a polyelectrolyte to silica,^36^ which was attributed to the replacement of surface-bound water with polymer train segments. Furthermore, the adsorption of three different surfactants onto kaolin clay has previously been studied using relaxation NMR,^37^ where a decrease in the relaxation rate was observed from the surfactant blocking water access to the paramagnetic surface.

Similar to the study on kaolin,^37^ it is likely in the case of these experiments that the *R*_sp_ reduction is induced due to the blocking of the Al-rich TiO_2_ surface by SHMP extending into the solvent. The most abundant aluminum isotope (^27^Al) is a quadrupolar nucleus and can cause enhancements in relaxation rates of bound solvent molecules at the colloid surface.^25^ Additionally, the current authors previously found an increase in the *T*_1_ relaxation rate of similar TiO_2_-SHMP systems after washing,^3^ which was assumed to be due to the exposure of the Al sites at the particle surface (although no comparison was made for TiO_2_ without SHMP). This agrees with the data reported in Figure 1, as here the surface Al solvent sites are blocked with increased SHMP adsorption. Interestingly, the reduction in *R*_2sp_ does not appear to reduce functionally the same as the adsorption isotherm (with a fairly monotonic decrease shown). Similar deviations in exact trends have been previously observed with polymer adsorption onto colloidal silica and alumina,^49,51^ in those cases due to the ^1^H NMR relaxation data being influenced more by the polymer train segments.^51^ Currently, there is likely a complex interplay between adsorbed polyphosphate enhancing the relaxation rate of surface-bound water, while also acting to block solvent from surface sites, resulting in an overall *R*_2sp_ reduction. Depending on the layer density, the changes will deviate (similar to the effect of adsorbed polyelectrolytes on ^1^H NMR relaxation^36^). Nonetheless, the plateau of the measured *R*_2sp_ agrees with the onset of the plateau in the adsorption isotherm at ∼600 ppm. Above this concentration, it can be concluded that further addition of SHMP in the bulk solution does not affect the adsorbed polyphosphate layer because solvent relaxation NMR is sensitive to the surface-bound water at the SHMP-particle interface.

**Figure 2:**
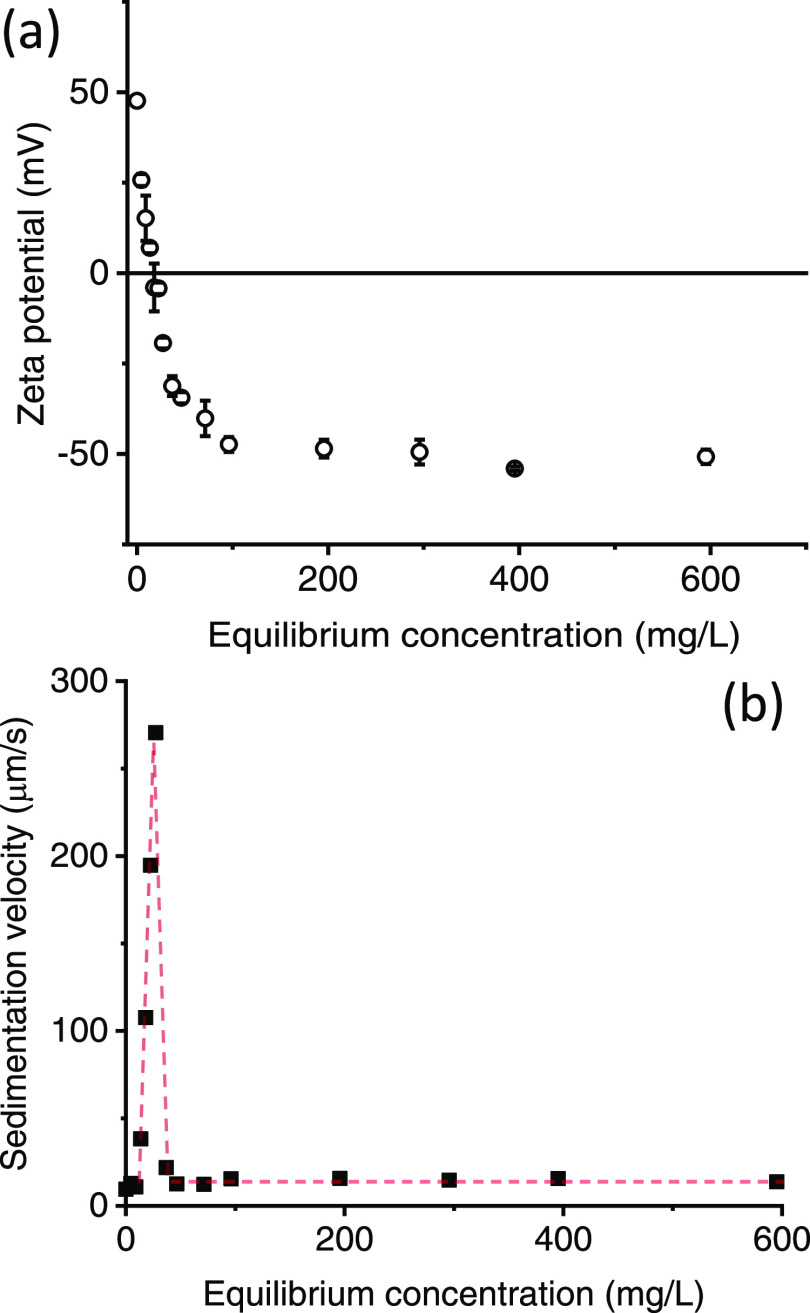
Changes in (a) titania ζ potential and (b) centrifugal sedimentation velocity with adsorption of SHMP. The dashed line in (b) is a guide to the eye.

It is also noted that lower equilibrium concentrations of SHMP (<200 ppm) could not be used for the NMR study, as dispersion stability problems were encountered. Although it would be useful to explore the changes in relaxation rates for lower SHMP surface coverage, this is not possible if dispersions become unstable and settle, as this leads to unrepresentative relaxation rates (and noting *T*_2_ measurements take ∼3 to 5 min to complete). As evidence of the changes that occur with low concentrations of SHMP, Figure 2a shows the decrease in the titania ζ potential with SHMP adsorption. The potential is at around −50 mV for equilibrium SHMP concentrations between 200 and 1000 ppm at pH 4, while at equilibrium SHMP concentrations <100 ppm, it varies rapidly from −50 to +50 mV, with the i.e.p. around 20 ppm SHMP. The change in surface charge is likely a result of patchy surface coverage for low SHMP concentrations, and ultimately this leads to particle aggregation and settling, where significantly enhanced centrifugal settling velocities were also measured within the region of i.e.p. (see Figure 2b). This instability meant that no NMR measurements could be taken within this initial concentration region.

### 3.2 Surface Area Comparison of Milled Titania without SHMP Adsorption, Using BET and NMR

**Figure 3:**
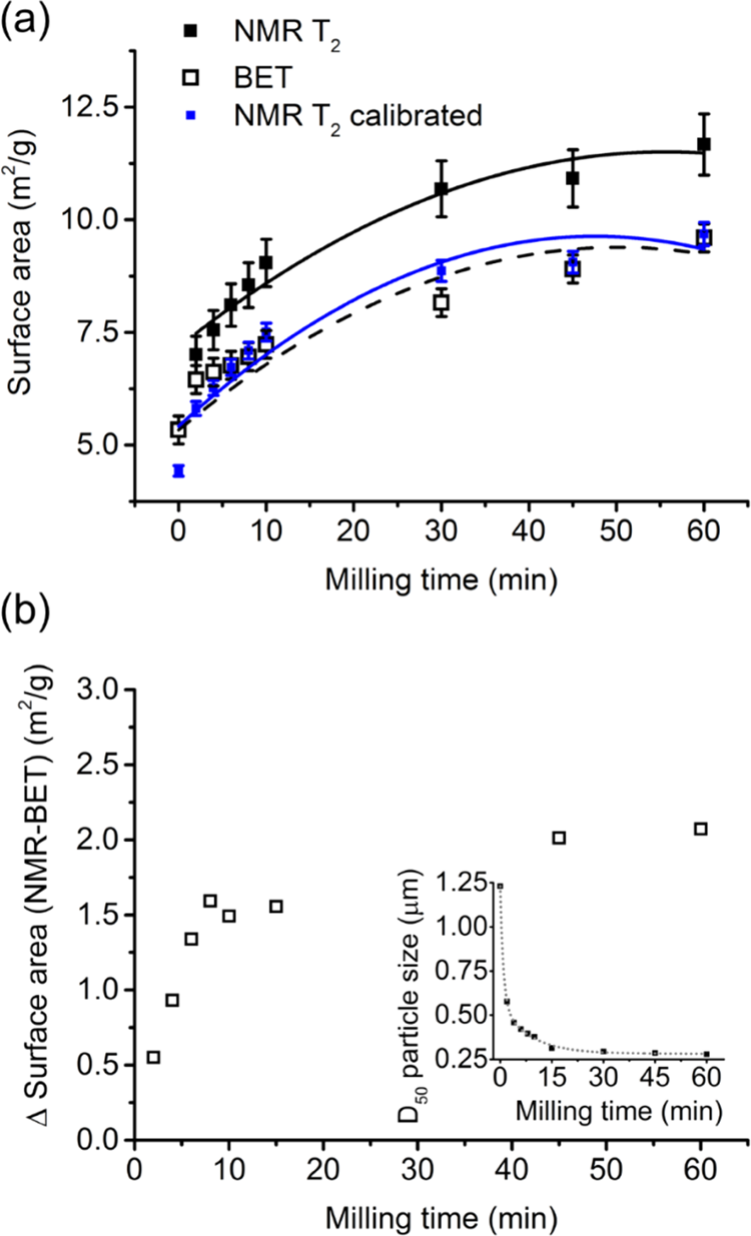
(a) Titania surface area versus milling time measured by BET and *T*_2_ relaxation NMR. NMR surface areas were calculated using both the *K*_a_ value calibrated from the 0 min milled BET surface area (black squares) as well as the average *K*_a_ value from all milling times (blue squares). Error bars represent the maximum BET variation, with the NMR error calculated from *K*_a_ ± this variation. Polynomial fits are shown to guide the eye. (b) Change in surface area between the two techniques when the NMR is not calibrated. Inset is the *D*_50_ particle size with milling time.

As well as being used to study the influence of SHMP on unmilled titania, relaxation NMR was also considered as a potential at-line technique to track surface area changes with milling. It is important to note that the NMR surface area calculation requires the derivation of a material-specific relaxation constant, *K*_a_, which was initially calculated using the BET surface area of the 0 min sample (with the *T*_2_*K*_a_ = 0.0130 g/(m^2^·ms)). Using this value, Figure 3a presents the measured surface area versus milling of titania-only samples (without SHMP), as measured from the NMR (using wet dispersions) and BET (using the dried powders). Here, the NMR data using this *K*_a_ derived from the 0 min sample (“NMR T_2_”, black squares) is shown in comparison to an improved calibration, using an average *K*_a_ value across the samples (“NMR T_2_ calibrated”, blue squares).

After 60 min of milling, the TiO_2_ NMR surface area is ∼11.7 ± 0.7 m_2_/g (giving a spherical equivalent particle size of 121 ± 7 nm), where the system *K*_a_ was estimated from the zero minutes milling time and assumed constant (black squares). The BET surface area was a slightly lower value of 9.6 ± 0.24 m^2^/g (correlating to a spherical equivalent primary particle size of 148 ± 4 nm). Both spherical equivalents underpredict measured median (*D*_50_) particle sizes, as measured by laser diffraction (inset, Figure 3b), but such equivalence must be made with caution. It is known that pigment titania tends to form nonspherical elongated rodlike particles, with a higher surface area-to-volume ratio^3,45^ where laser diffraction will represent more maximum length dimension. Also, there is evidence that excessive milling may form very fine <20 nm satellite particles from breakage, which may increase the surface area significantly.^3^ The fact that the relaxation NMR surface area measurements are higher than BET may partially support this, as it is a dispersion technique where any satellite particles will occupy their largest wetted surface area (such particles will conversely adhere onto larger primary particles in dry powder BET).

Nonetheless, it is likely that changes in satellite particle dispersion alone cannot account for such differences in the surface area measurements, and it should be noted that the NMR technique is sensitive to changes in surface chemistry as well as surface area. To investigate further, the difference in the surface area (ΔSA) between NMR and BET measurements is shown in Figure 3b, where the ΔSA steadily increases until about 15 min of milling and then begins to plateau (although the plateau value is not directly reached within the period). Interestingly, particle size changes measured by laser diffraction (inset) also follows this general trend, with size reduction plateauing post 15 min of milling as well. Therefore, system changes within the first 10–15 min of milling account for most differences in surface area measurement.

It has been assumed that changes in surface chemistry are minimal with milling time. However, Austin et al.^45^ recently reported changes in surface alumina concentration of pigment titania with an increase in milling time, which also stabilized after 15 min of milling in similar systems. As previously discussed, aluminum causes enhancements in the relaxation rate of bound solvent molecules at the colloid surface and so small changes in the surface concentration of Al are likely to have a considerable impact on the calculated *K*_a_ for Al-doped TiO_2_. Thus, the *K*_a_ at each milling time was measured, and a mean *K*_a_ was calculated by using the BET surface area for each milled sample, which was then used to recalculate the NMR surface area for an improved calibration (as also shown in Figure 3a, blue symbols). After this correction, the NMR and BET agree within the measurement error and illustrate that relaxation NMR can be used as a rapid at-line technique to determine particle surface area for Al-doped TiO_2_ with milling, despite changes in surface aluminum concentrations.

### 3.3 Adsorption and Degradation of SHMP after Milling Probed by XPS

XPS was used to probe the influence of milling time on SHMP adsorption and potential degradation, to gain a more detailed understanding of the dispersant bonding at the particle surface. The survey spectra for unmilled TiO_2_ with SHMP adsorbed and the separate reference spectra of SHMP and TiO_2_ are given within the SI (Figure S3). The main signals in the spectrum were Ti, O, P, Al, Na, and C, arising from either the particle surface Al-doped TiO_2_, polyphosphate SHMP or bonding between the particle and dispersant in the form of Al–O–P or Ti–O–P bonds.

**Figure 4:**
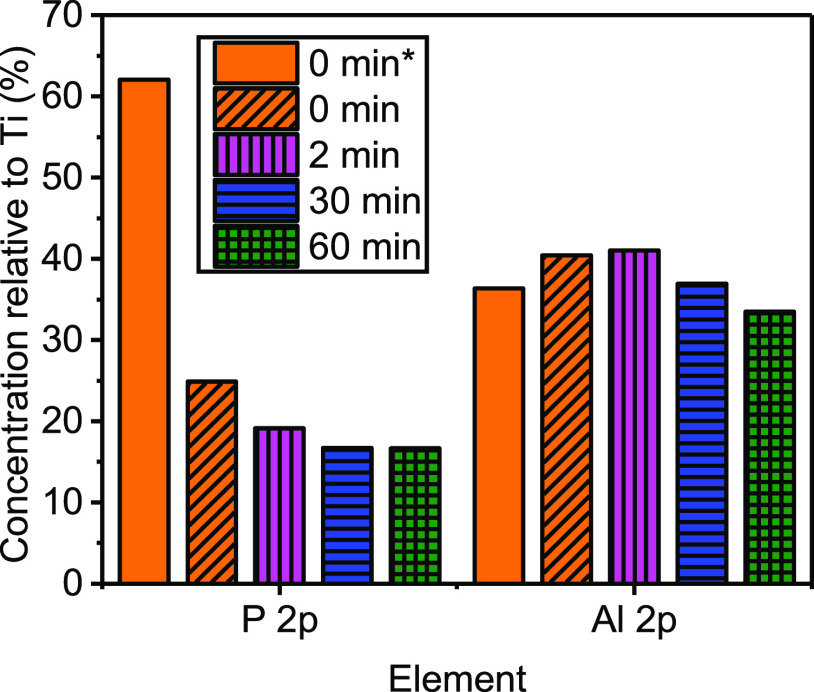
Elemental quantification of milled samples (washed) and unmilled (washed and unwashed*) obtained from XPS analysis, shown as a relative concentration to Ti (%) (Ti = 100%) for the P 2p and Al 2p compositions.

Figure 4 presents the elemental quantification of phosphorus (P 2p) and aluminum (Al 2p) obtained by XPS for milled samples at different times, along with an unmilled sample that was dried without excess washing (0 min*). The data was normalized to the relative content of Ti as a percentage (which was approximately constant with time). There is a clear decrease in the P 2p concentration with an increase in milling, which suggests the energy of milling is leading to either fractional removal of the SHMP at the particle surface or partial dispersant degradation. Regardless of the origin of this effect, it is clear that elongated milling times are causing reductions in the adsorbed SHMP layer.

Also shown in Figure 4 is a large decrease in the P 2p concentration of the unmilled TiO_2_ after centrifugal washing, which indicates that there is a considerable portion of physisorbed or weakly bound SHMP that is initially removed through the washing stage, at least at pH 4. It is also noted that this fraction removed would also include some nonadsorbed dispersion SHMP that crystalized on particle surfaces upon drying, as it was present in excess conditions. Nevertheless, it is clear that phosphorus is still present to a significant degree after washing, which may be assumed to be mostly from SHMP that is bound strongly by chemisorption at pH 4. Thus, it would be probable that the TiO_2_ remains stable even after washing, so long as the surface charge remains repulsive and not patchy. Similar mixtures of strong and weakly bound dispersants have been found previously on industrial TiO_2_ pigment dispersions, although the industrial process itself does not contain an intermediate washing step to remove the weakly bound fraction^3^ (as washing occurs downstream after the application of all inorganic treatments).

Additionally, Figure 4 presents the relative aluminum concentration (Al 2p) with milling time, where the surface coverage as an absolute percentage is also given within the SI (Table S3). Interestingly, there is no consistent trend in Al surface changes as milling progresses. This result is counter to expectations from the milling data without SHMP present, where it was assumed that aluminum surface increases may correlate to shifts in the NMR relaxation rate at longer times (see Figure 3). It would also be seemingly incongruous with the mentioned work of Austin et al.,^45^ who studied the effect of milling on very similar pigment titania systems (again without SHMP present). They found that Al concentrations increased from ∼5.7 to 9.5% after 60 min of milling and observed a shift in the i.e.p. for Al-doped titania to pH 8 after milling (toward that likely for an alumina surface). While the initial levels are very similar to those observed in the present case (Table S3), there is a clear disparity in the effect of milling on surface aluminum changes. It is hypothesized that the critical difference is the effect of the SHMP on lubricating particle surfaces with milling, reducing changes to the particle crystal surfaces. In fact, in other work with pigment titania and SHMP, Austen et al.^5^ found lubrication effects of SHMP also led to lower viscosity dispersions that become most evident at long milling times and was dependent on the extent of dispersant surface coverage. In this study, while the lubrication effect of SHMP itself was assumed to be fairly constant during milling, the viscosity of dispersions without SHMP increased, which was attributed to the formation of fines and secondary aggregation when no dispersant was present.

**Figure 5:**
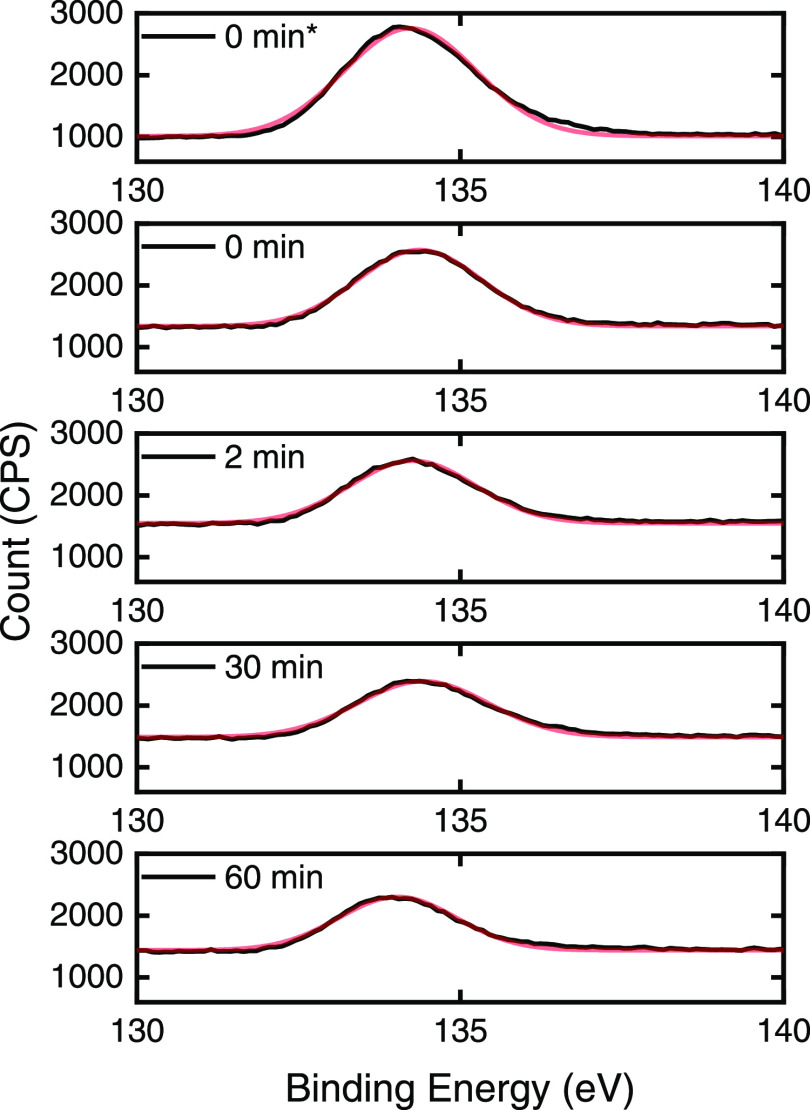
P 2p band for milled and unmilled (washed and unwashed*) titania samples fitted to a gaussian distribution shown in red.

To further investigate whether changes evident with dispersant may imply any chemical degradation, or just partial removal with milling time, Figure 5 gives the P 2p band for milled TiO_2_ samples that have been fitted to a gaussian distribution. The peak full width at half-maximum (FWHM) and central positions are given within the SI (Table S4). The fitted P 2p peak widths show little to no change with an increase in milling time from 0 to 60 min, within experimental error. The FWHM can be a useful indicator of chemical state changes or physical influences, for example, broadening of the FWHM can be caused by a variation in the number of chemical bonds contributing to the peak shape, alteration in the sample condition due to X-ray damage, or charging across the surface.^52^ Hence, if milling does cause SHMP chemical degradation, it is likely not affecting a considerable portion of the dispersant coating.

Nevertheless, also shown in Table S4, is a small shift in the binding energy of the P 2p band from 0 min of milling (134.46 eV) to 60 min (133.95 eV). Xi and Liu^17^ found the P 2p for pure SHMP was 134.1 eV and SHMP treated calcite was 133.9 eV, both in agreement with the results observed presently, implying the slight reduction in milling time may be the result of partial SHMP loss, rather than degradation. In fact, while there is strong literature evidence of binding energy values being lower for smaller polyphosphate compounds^53,54^ and breakage of P=O bonds, for example,^55,56^ reported shifts in binding peaks are normally much greater than the 0.5 eV shift recorded here (between 0 and 60 min samples). Therefore, it is important to quantify if there are any other chemical changes that may be observed in the survey scan from changes in polyphosphate structure.

**Figure 6:**
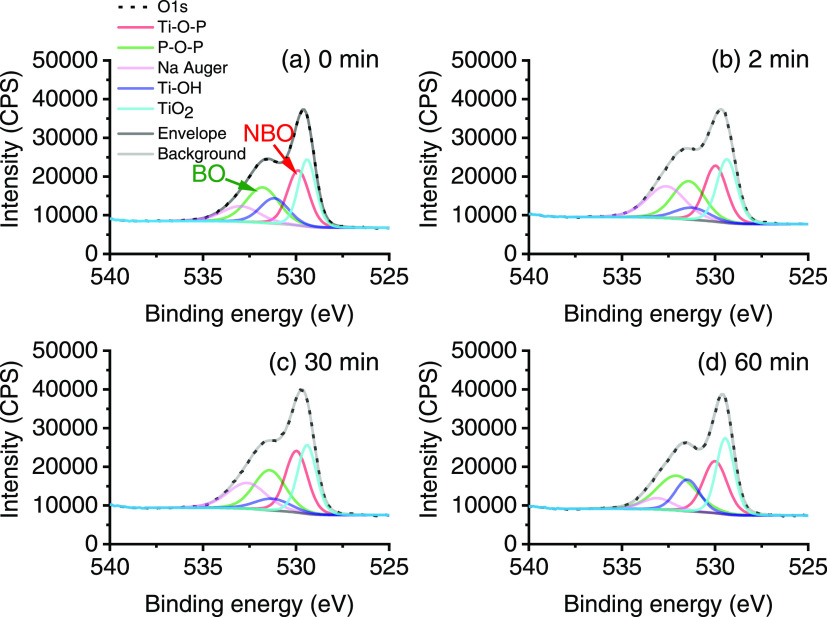
O 1s peaks with fitted components of bridging oxygen (BO) and non-bridging oxygen (NBO) for titania with adsorbed SHMP after milling for (a) 0 min, (b) 2 min, (c) 30 min, and (d) 60 min.

To consider other bands, the O 1s spectra were characterized and peaks deconvoluted, as given in Figure 6. The O 1s spectra for unmilled Al-doped TiO_2_ and pure SHMP were used as a reference and are given within the SI (Figure S4). The O 1s XPS signals were curve-fitted using assignments for rutile oxygen and hydroxyl groups arising from both titania and alumina. The fitted rutile oxygen component was found at 529.5 eV with an FWHM of 1.26 eV, in agreement with the literature for Al-doped TiO_2_ studies^57−59^ and the hydroxide component at 531.6 eV with an FWHM of 2.54 eV and consistent with the literature for Al–OH species^16,57^ and Ti–OH species.^57,59,60^ The O 1s signal for pure SHMP (Figure S4b) was fitted with three components, arising from P=O and P–O–P bonds within the SHMP chain (531.3 eV, FWHM 1.45 eV) and Na Auger peaks at 533.0 and 536.3 eV, and was also in good agreement with the literature.^61^

XPS analysis of phosphate glasses was first reported in the 1970s, and several studies state that the O 1s signal can be fitted to two different components.^62^ The first component which resides at a higher binding energy consists of bridging P–O–P oxygen atoms and is often denoted as BO (bridging oxygen), and the second component is a mixture of terminal oxygen atoms (P=O and P–O–M) and often called non-bridging oxygen (NBO) which is found at a lower binding energy.^62−64^ The latter NBO peak can be fitted to a single distribution since the chemical shift between the P=O and P–O–M signals is small.^62^ However, for the milled samples analyzed in Figure 6, the O 1s peaks were fitted to five components (see Figure 6a) consisting of an Na Auger peak (due to the presence of Na in SHMP), NBO peak and BO peak, where it is assumed that although the P–O–M peak would consist of M = Ti and Al in varying amounts, the shift in signal is expected to be small and has been neglected. Also present in the sample will be TiO_2_ (where previous studies have fitted the O 1s peak with BO, NBO, and metal oxide peak^64^) and TiOH from unbound SHMP sites, fitted using components found in the reference spectrum of Al-doped TiO_2_ (Figure S4a).

Previous studies^62−64^ have shown that it is possible to distinguish polyphosphate chain length in XPS by calculation of the ratio between the BO and NBO peaks used for fitting the O 1s peak. Hence, the intensity ratio between BO and NBO was calculated for Al-doped TiO_2_ with adsorbed SHMP at different milling times, as found in Table 1.

**Table 1:** O 1s Components for the Bridging Oxygen (BO) (P–O–P bonds) and the Non-Bridging Oxygen (NBO) (Ti–O–P and P=O Bonds) for SHMP Adsorbed Titania as a Function of Milling Time^a^

milling time (min)	BO O 1s (eV)	NBO O 1s (eV)	Δ*E* (eV)	FWHM BO O 1s (eV)	FWHM NBO O 1s (eV)	ratio O 1s BO/NBO
0	531.79	529.88	1.91	2.07	1.36	0.80
2	531.40	529.97	1.43	2.03	1.41	0.82
30	531.41	529.97	1.44	2.02	1.40	0.79
60	532.07	529.99	2.08	2.50	1.52	0.83

a BO and NBO O 1s peak positions (eV), difference between these peaks, Δ*E* (BO-NBO), and intensity ratio (BO/NBO).

In the present work, the O 1s NBO component peak was essentially invariant between milled samples (529.9–530.0 eV) with the BO component varying slightly more (531.4–532.1 eV), where the BO peak agrees well with the binding energy of SHMP adsorption onto kaolinite.^16^ To gain an insight into the influence of milling time on the position of the BO peak, the distance between the NBO and BO was calculated (Δ*E*). This value varied for the milled samples between 1.4 and 2.1 eV, although with no clear trend with respect to milling time. Previous work^62^ has shown that ZnO content (from the milling media) influenced the BO peak for different polyphosphate chain lengths, where the value of zinc pyrophosphate was 1.43 eV and that for zinc metaphosphate was 1.67 eV.

The intensity ratio BO/NBO calculated in this work was also essentially unaffected by milling time, with other studies using this as an indicator of phosphate chain length,^62^ which could be a further indication that little phosphate hydrolysis occurs from the energy of milling. Nonetheless, there is a substantial increase in the BO FWHM observed for the 60 min milled sample, which suggests that more chemical environments are present. Such changes may indicate the potential for very extended milling times to induce some partial hydrolysis, resulting in the removal of a greater proportion of the polyphosphate.^3^ Therefore, there is some evidence of minor chemical changes in the SHMP at excessive milling times, although likely not to the majority of the SHMP layer. It may be after extreme milling, some crystal breakage is occurring, exposing new surface chemistry. Future research into the XPS valance band region is recommended, where some previous studies have shown a greater ability to differentiate phosphate compounds and their oxides, compared to information that can be identified by the core levels.^65,66^ It is also noted that potential changes to polyphosphate chemical states could result from coordinate bonding to leached titania or aluminum ions in solution^12^ generated through milling. Such changes may also alter adsorption behavior. While there was no discernable change in surface Ti signal with milling time, and the Al concentration was also not consistently affected by milling (although, presenting some variation) as discussed in relation to Figure 4, no direct measurements of ion leach rates were able to be made. Future work will assess both solid surface and aqueous concentrations of metals to aid fuller understanding of the effects of milling on polyphosphate structure and adsorption behavior.

### 3.4 ζ Potential and Relaxation NMR of Milled Suspensions

**Figure 7:**
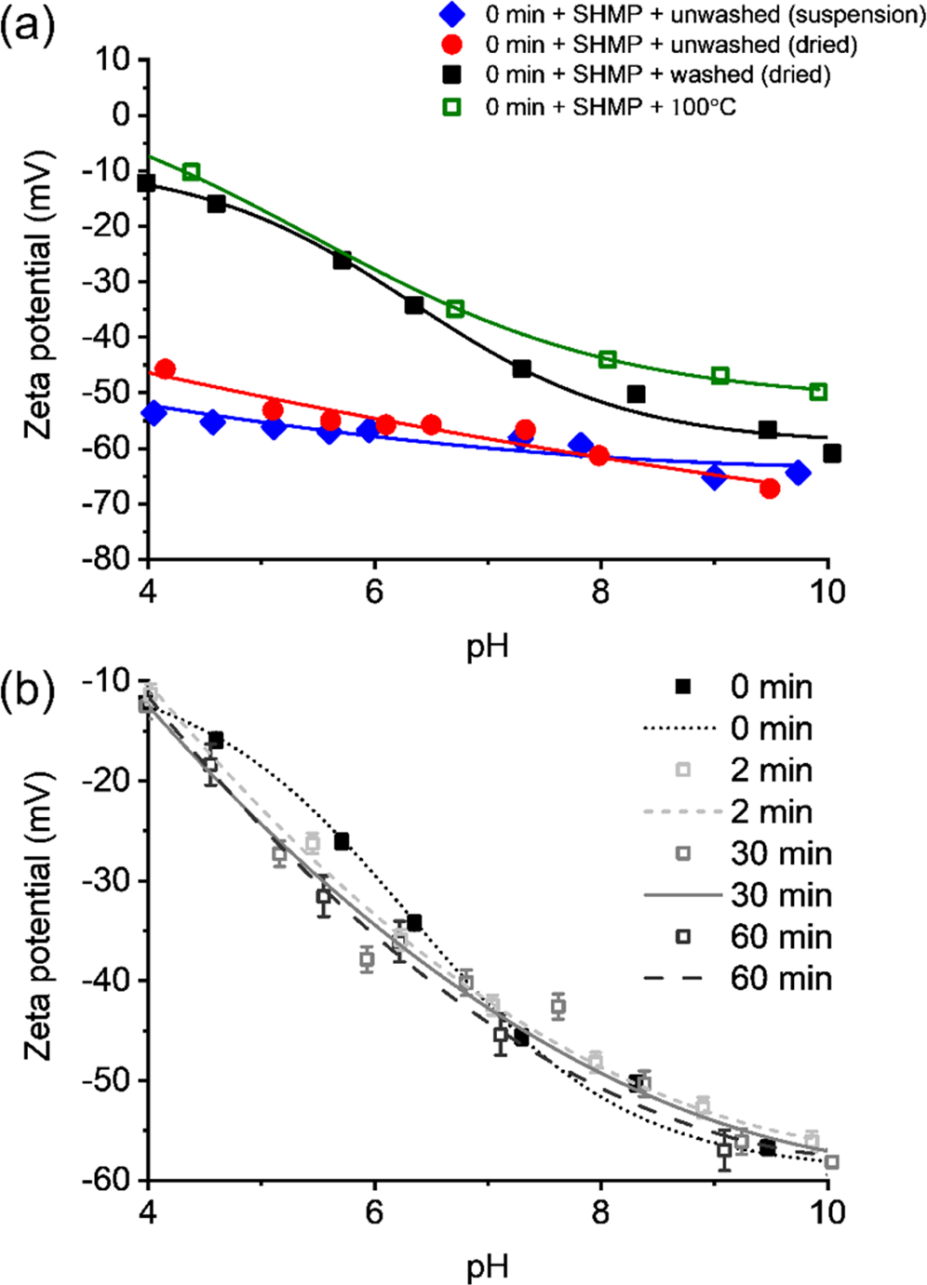
ζ potential of Al-doped TiO_2_ with adsorbed SHMP under different processing conditions. (a) Unmilled dispersions with and without centrifugal washing and drying, as well as a sample heated to 100 °C. (b) Charge as a function of milling time. Lines are a guide to the eye.

Figure 7a shows ζ potential as a function of pH after SHMP adsorption onto Al-doped TiO_2_. At all pH values, the pigment ζ potential in the presence of polyphosphate is considerably more negative compared to the surface charge of the bare pigment (Figure S1).^15,43^ For example, at pH 4, the pigment surface charge is approximately +30 mV, and after SHMP adsorption, it is around −50 mV, prior to washing. Importantly, after centrifugal washing, the ζ potential becomes much less negative, consistent with the partial removal of polyphosphate evidenced with XPS. The ζ potential still remains negative after washing due to the remaining SHMP being chemisorbed through Ti–O–P bidentate complexation^43^ and any remaining electrostatically physisorbed SHMP. It also appears from the ζ potential measurements that the drying procedure did not affect the TiO_2_, as the surface charge is unchanged across the pH range studied for the dried and redispersed suspensions. Additionally, the act of heating washed SHMP-TiO_2_ suspensions to 100 °C did not cause significant hydrolysis or further SHMP removal, at least for the timeframe studied (1 h), as the ζ potential was again relatively unchanged.

Figure 7b presents the ζ potential for washed and dried pigment after adsorption of SHMP, for different milling times. The measured i.e.p. for milled samples was at ∼pH 3–3.5 (data not shown) and agrees for polyphosphate adsorption to Al-doped titania surfaces.^5,43^ Interestingly, there appears to be minimal change in ζ potential with milling time (although there is a slight shift to more negative potentials at lower pH values). Generally, the lack of change is indicative of well-coated surfaces where the SHMP is available in excess. While XPS data showed clear evidence for partial removal of the SHMP with milling time, there is still sufficient dispersant coating to generate strong electronegative surfaces. The slight increase in the magnitude of the ζ potential at long milling times may be suggestive of the effect of milling on pigment dispersion and a reduction in agglomerates. A similar but much more dramatic increase in ζ potential magnitude has been previously observed by the current authors with industrially milled pigment samples.^3^ As milling occurs, de-agglomeration of the suspension may expose further surface sites, allowing more SHMP to adsorb. The difference from the previous samples is from the fact that only partial SHMP adsorption occurs initially within the industrial process, leading to much more significant effects from dispersant mixing. Indeed, another study by Austin et al.^5^ has indicated that differences in milled ζ potentials of TiO_2_-SHMP suspensions are nullified with high ratios of dispersant coverage.

To further probe dispersion characteristics, particle size measurements were taken for the same systems over time (see the SI, Figure S5). A clear reduction in particle sizes is evident, highlighting again that sufficient SHMP remains adsorbed onto the surface to reduce particle aggregation and re-agglomeration with milling (with most changes occurring within the first 30 min). Gaussian peak deconvolution was also used (Figure S5b) to link size distributions more explicitly to changes in particle aggregates, with a clear increase in the primary particle peak and inverse reduction in the aggregate peak over time.

**Figure 8:**
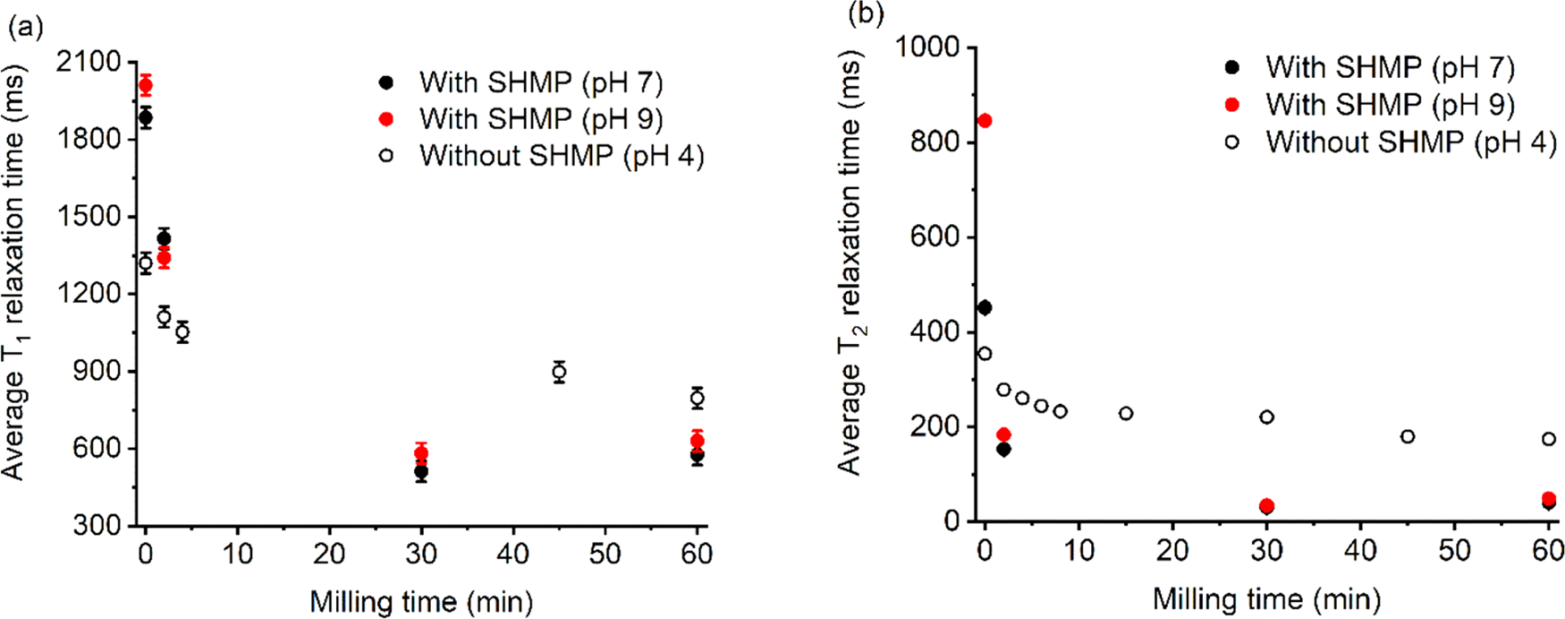
Relaxation NMR (a) *T*_1_ and (b) *T*_2_ for Al-doped titania dispersions with and without SHMP after milling for different times.

To understand the effect of milling more completely on SHMP coverage, *T*_1_ and *T*_2_ NMR relaxation measurements were performed for milled Al-doped TiO_2_ both with and without SHMP adsorbed, as presented in Figure 8 in terms of the total relaxation times (rather than the relaxation rate). Both *T*_1_ and *T*_2_ were measured, as although they will ostensibly shift in a similar response to changes to surface area or chemistry, the magnitudes of the shifts are different, with T_2_ potentially being more sensitive to surface area changes.^3,19,25^ Here, it is noted that the *T*_2_ relaxation data for systems without SHMP is the same as that used for surface area analysis in Figure 3, while for systems with SHMP, the pH was adjusted to both 7 and 9 to mimic industrial process conditions. Tests were not conducted at pH 4 for TiO_2_-SHMP systems, as this was too close to the i.e.p. (Figure 7) leading to some aggregation. Also, previous work by the current authors has shown that changes in particle surface charge can have a critical effect on the measured relaxation rate,^26^ especially in high electrolyte conditions. In this case, as the change in ζ potential was only minor in the region of pH 7–9 (see Figure 7), there is correspondingly little discernible difference in the measured *T*_1_ or *T*_2_ relaxation times between either pH for the TiO_2_-SHMP systems.

For short milling times (<5 min), the relaxation time is faster for the bare particle surface without SHMP, which is assumed to be due to aluminum present on the particle surface. As discussed, aluminum (^27^Al) is a quadrupolar nucleus and can cause enhancements in the relaxation rates of bound solvent molecules at the colloid surface.^25,37^ In correlation, the adsorption of SHMP onto unmilled titania (*t* = 0 min) appears to result in longer relaxation times than the bare surface, as SHMP extends into the solvent and surface-bound water molecules are blocked from the Al-rich TiO_2_ surface. Similarly, Totland et al.^37^ observed a decrease in the NMR relaxation rate after the adsorption of a surfactant onto kaolin clay, again due to the surfactant blocking water access to the paramagnetic surface.

More importantly, Figure 8 indicates that there is a crossover in the relaxation times for longer milling times (>5 min) where the bare particle surface has a longer relaxation time than the SHMP adsorbed particle surface (and is similar for both *T*_1_ and *T*_2_). Given the clear enhancement to the relaxation rate from surface aluminum with respect to SHMP, the exact reason for this crossover as milling occurs is not known exactly, and it is most likely due to a combination of factors. Some of the changes may be from differences in surface area changes with milling times for particles with or without SHMP, with a decrease in relaxation time for SHMP being attributed to a greater surface area increase compared to without SHMP. However, the surface area of a number of TiO_2_-SHMP samples was not notably different from those without dispersant (see data presented in ref (5)), which is consistent with previous work on milled minerals with dispersants.^67^ The fact that the samples without dispersant were measured at pH 4 and so are strongly cationic may also have contributed to differences; although the ζ potential magnitude is similar to the TiO_2_-SHMP samples at pH 7–9, and such effects would be evident at all sample times. Thus, it is more likely that surface chemistry effects are the cause of the crossover.

It is also noted that the similar trends between *T*_1_ and *T*_2_ measurements appear in contrast to previous industrial sample measurements of similar systems, where the *T*_2_/*T*_1_ ratio showed an inflection point at intermediate milling times.^3^ In this case, it was not expected that the SHMP was at equilibrium adsorption upon commencement of milling, with the inflection correlating to a point of full monolayer formation. In the current case, as milling was performed after full SHMP adsorption, the fact that *T*_1_ and *T*_2_ essentially show identical relationships over time may correlate to a monolayer being always present. As both ζ potential and size data also indicate good dispersion stability, this further suggests that sufficient SHMP coverage remains.

Nevertheless, XPS data (Figure 4) did show a clear reduction in the adsorbed SHMP levels with an increase in milling time, which was thought to be due to polyphosphate degradation from milling energy leading to partial removal of weakly SHMP from the surface (while changes in the chemical nature of the coating were less clear). This reduction is consistent with previous data from the current authors,^3^ which also showed partial removal of SHMP from industrially milled titania samples after centrifugal washing. In this case also, there was a measured increase in the *T*_1_ relaxation rates of washed samples that was apportioned to increased exposure to surface aluminum sites. It appears, therefore, that there may be a type of dual enhancement scenario where partial removal of SHMP as milling progresses (and potential changes in surface structure) leads to faster relaxation times than either the pure Al-doped titania surface or that from an excess SHMP coating. This enhancement appears even though evidence would also indicate sufficient coverage remains for good dispersion stability. Future work looking in more detail at SHMP over time (e.g., with valance band XPS) may aid in elucidating whether it is merely SHMP coverage or molecular structure that leads to the considerable increase in relaxation rate. Nevertheless, it is emphasized that this data also proves that relaxation NMR can be a valuable technique in tracking the extent of milling over time and could be applied as an at-line technique industrially. Careful considerations must be taken in how best to calibrate such a system for any surface area monitoring application, and correlations are required from mixed suspensions that are as close to their industrial counterparts as possible.

## 4 Conclusions

In this study, changes in sodium hexametaphosphate (SHMP) dispersant adsorbed onto aluminum-doped titania were investigated for samples undergoing wet milling (corresponding to industrial pigment production). A critical research question was whether the milling energy led to degradation in either the chemical structure of the dispersant or overall surface coverage, which may result in samples of reduced dispersion at long milling times or high mill rates. Relaxation proton NMR was also utilized as a potential at-line technique to monitor the extent of milling, including changes to surface area and surface chemistry, while XPS was used primarily to consider the dispersant structure.

In terms of XPS, results showed that considerable amounts of weakly adsorbed SHMP could be removed with washing, and the level of dispersant removal increased with milling time, highlighting the destructive effects of sustained high-energy milling. Nonetheless, washed samples continued to display strongly negative ζ potentials, indicating sufficient chemisorbed dispersant remained for good titania stability. Additionally, particle size distributions evidenced high dispersion levels, with a continual reduction in the aggregate peak over time inferring good SHMP coverage. Also, despite the continual removal of SHMP, detailed XPS analysis did not reveal significant chemical changes to the dispersant, although increases in the bridging oxygen (BO) FHMO suggested some chemical degradation was occurring at excessive milling times. Additionally, there was no evidence of changes in surface aluminum content with milling, in contrast to the assumed behavior without dispersant, likely due to dispersant lubrication effects.

Relaxation NMR revealed a number of important aspects related to surface area and surface chemistry changes with milling time. Results with unmilled material indicated that dispersant adsorption could be tracked with pseudo-isotherms using the relative enhancement rate (*R*_sp_) although samples with low adsorbent density could not be tested due to dispersion instability around the isoelectric point. Interestingly, the *R*_sp_ decreased with surfactant, owing to partial blocking of the quadrupolar surface aluminum. Milled samples were also tracked with and without dispersant to understand how well the technique could be used as an industrial at-line method. Very accurate calibrations of milled surface area were possible from either *T*_1_ or *T*_2_ relaxation data for systems without dispersant. Behavior was considerably more complicated for systems with SHMP, where there appeared an interplay between the dispersant surface coverage and relaxation enhancement from the surface aluminum. As dispersant adsorption density reduced due to the milling, the relaxation rate was enhanced at intermediate times, with respect to either samples without dispersant or high surface coverage dispersant suspensions. It was unknown whether these changes were due simply to reduced surface coverage or partial chemical degradation of the dispersant, and work is suggested into the use of valance band XPS to gain a clearer insight into the chemical state of the dispersant. Despite these complexities, this investigation highlighted that relaxation NMR could be used as a real-time technique to monitor the extent of industrial milling processes, reducing issues with overmilling, so long calibrations can be achieved on systems close to their industrial counterparts. Indeed, in future, it should be possible to fully deconvolute both surface area and surface chemistry effects in the process, potentially making relaxation NMR a very powerful analytical technique.

## Supporting Information Available

Zeta potential versus pH for unmilled Al-doped TiO_2_ (Figure S1); summary of Langmuir adsorption fitting procedure and adsorption constants (Table S1), along with linear data fit (Figure S2) and literature comparison (Table S2); XPS survey spectra (Figure S3), resolved Al concentrations (Table S3) and fitted P 2p band data with milling time (Table S4) for Al-doped TiO_2_ with SHMP, as well as O 1s fitted peak deconvolution (Figure S4); and measured particle size distributions and peak fits versus milling time (Figure 5S) (PDF)
