# Supplementary material for: Analysis of Adsorbed Polyphosphate Changes on Milled Titanium Dioxide, Using Low-Field Relaxation NMR and Photoelectron Spectroscopy
Source: Langmuir. 2023 Apr 13;39(16):5697–709. doi: 10.1021/acs.langmuir.2c03416 (PMC10134495; doi:10.1021/acs.langmuir.2c03416)
Supplement: Supplementary file 1 — la2c03416_si_001.pdf [file la2c03416_si_001.pdf]

Electronic Supplementary Information (ESI)

Analysis of adsorbed polyphosphate changes on milled titanium dioxide, using low-field relaxation NMR and photoelectron spectroscopy

Laura N. Elliott[a,b], David Austin[a,b], Richard A. Bourne[a,c], Ali Hassanpour[a], John Robb[d], John L. Edwards[d], Stephen Sutcliffe[d], and Timothy N. Hunter[a,*]

[a]School of Chemical and Process Engineering, University of Leeds, Leeds LS2 9JT, U.K.
[b]Centre for Doctoral Training in Complex Particulate Products and Processes, University of Leeds, Leeds LS2 9JT, U.K.
[c]Institute of Process Research and Development, School of Chemistry, University of Leeds, Leeds LS2 9JT, U.K.
[d]Venator Ltd, Titanium House, Hanzard Drive, Wynyard Park, Stockton-on-Tees TS22 5FD, U.K.

[*]Corresponding author: t.n.hunter@leeds.ac.uk

Further Particle characterisation data

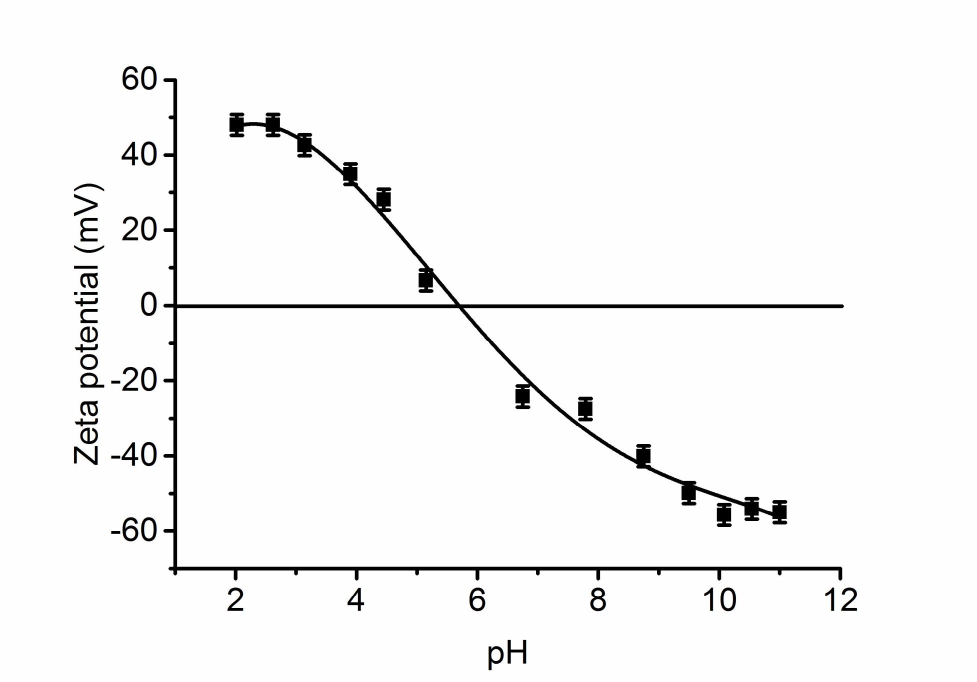

Fig. S1: Zeta potential of unmilled Al-doped TiO_2 in 1 mM NaCl background electrolyte.

Summary of Langmuir Isotherm method and fitting data

The Langmuir adsorption model was applied using the linear translation of the Langmuir isotherm, as shown in Eq. (S1). Here, C_e is the adsorbate equilibrium concentration (mg/L), q_e is the amount of adsorbate in the adsorbent at equilibrium (mg/g), q_m the maximum adsorption capacity (mg/g) and b is the Langmuir adsorption constant.

$$\frac{C_e}{q_e} = \frac{1}{q_m b} + \frac{C_e}{q_m} \qquad \text{Eq. (S1)}$$

The amount of SHMP adsorbed onto the adsorbent at equilibrium was calculated using Eq. (S2). Here, C_0 is the initial concentration of SHMP in solution (mg/L), m is the mass of adsorbent (g) and V is the volume of SHMP solution (L). In experiments, C_e is obtained from ICP-OES measurements.

$$q_e = (C_0 - C_e)\frac{V}{m} \qquad \text{Eq. (S2)}$$

A linear plot of C_e/q_e vs. C_e, as shown in Fig. S2, gives a gradient of $1/q_m$ and intercept of $1/(q_m b)$, thus allowing the Langmuir adsorption constant (b) and maximum adsorption capacity to be calculated (q_m), given in Table S1.

Table S1: Calculated parameters using the linearised form of the Langmuir equation.

Maximum adsorption, q_m (mg/g)	5.03 ± 0.5
Langmuir constant, b (L/mg)	0.034 ± 0.002

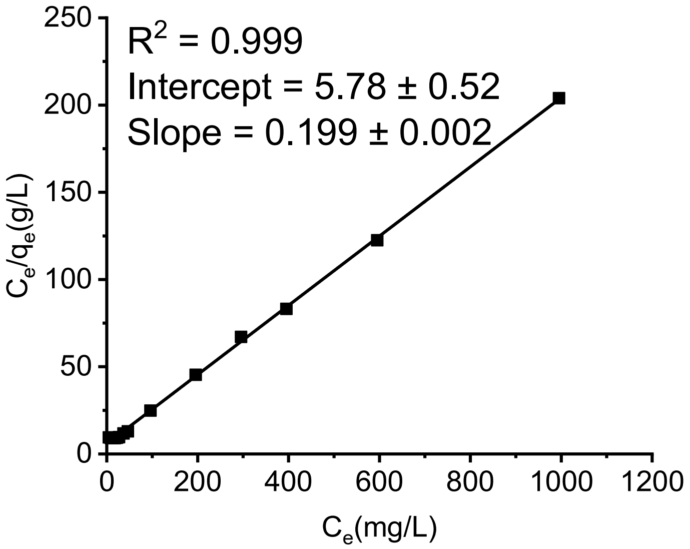

Fig. S2: Linear Langmuir plot of SHMP adsorption used to calculate the Langmuir isotherm parameters.

Table S2: Maximum adsorbed amount of polyphosphate onto Al-doped TiO_2 pigment using the Langmuir equation. Shown is a comparison to the adsorbed quantity found by Taylor et al. (2001)[1], with the differences in experimental conditions highlighted.

**Parameter**	**This experiment**	**(Taylor et al., 2001)**
BET surface area (m^2/g)	5.34	9.2
Polyphosphate chain length (n)	6	10-12
pH	4	4
Maximum amount of adsorbed polymer (mg/m^2)	0.95	0.56

[1] Taylor ML, Morris GE, Smart RSC. Polyphosphate interaction with aluminium-doped titania pigment particles. Colloids and Surfaces A: Physicochemical and Engineering Aspects. 2001;190(3):285-94. Citation Also appears in main manuscript.

Further XPS Data

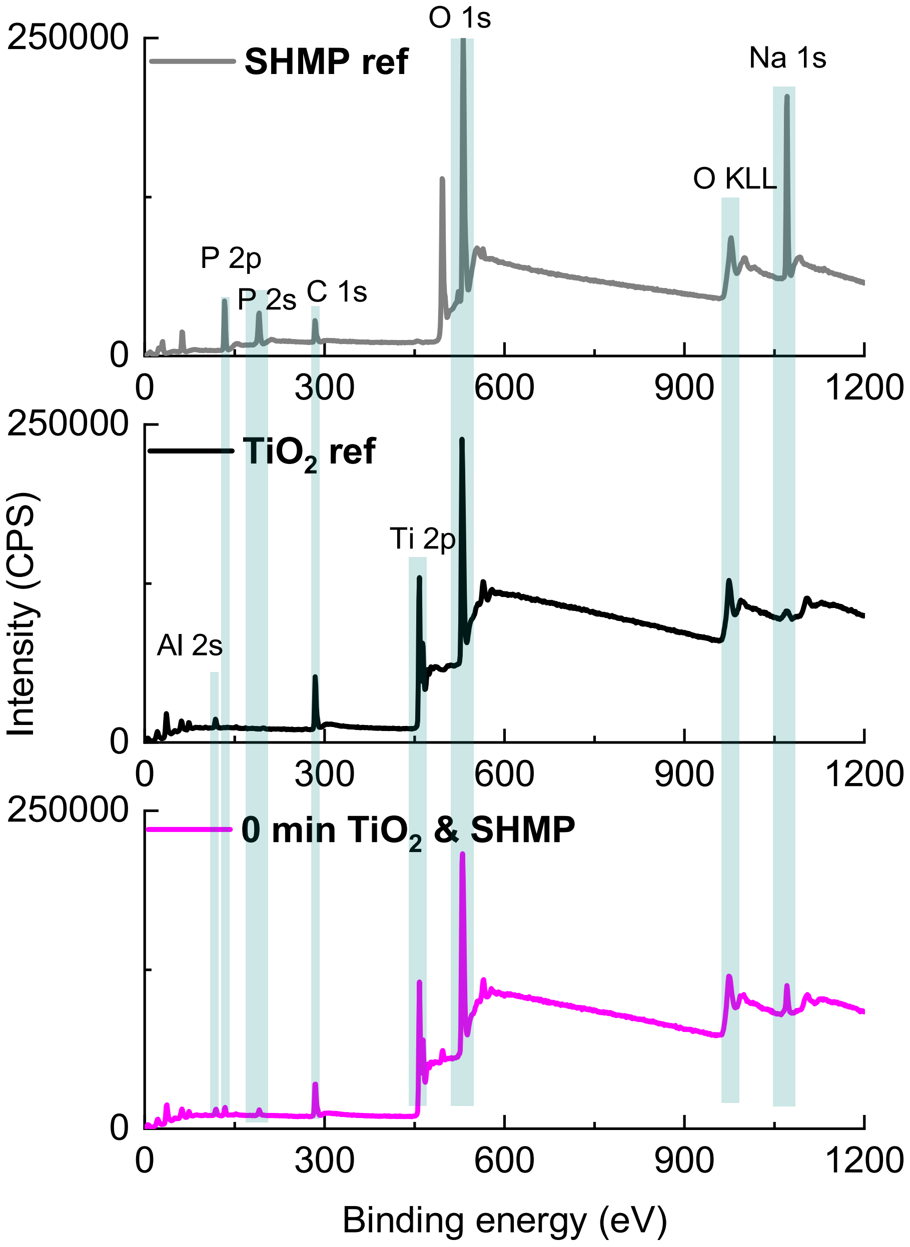

Fig. S3: XPS survey scans of SHMP reference, TiO_2 reference (no SHMP) and 0 min milled TiO_2 with SHMP adsorbed after washing.

Table S3: Absolute Al concentration (%) determined by XPS for Al-doped TiO_2 pigment in the presence of SHMP after milling.

Milling time (min)	Level of Al (%)
0	5.3
2	4.6
30	5.0
60	3.7
	Average (4.7)

Table S4: P 2p band fitted peak full width half maximum (FWHM) and peak position as a function of milling time for washed TiO_2 with SHMP.

Milling time (min)	FWHM (eV)	Position
0	2.21 ± 0.25	134.46 ± 0.02
2	2.21 ± 0.35	134.26 ± 0.03
30	2.32 ± 0.32	134.17 ± 0.02
60	2.15 ± 0.31	133.95 ± 0.02
Average	2.26 ± 0.28	-

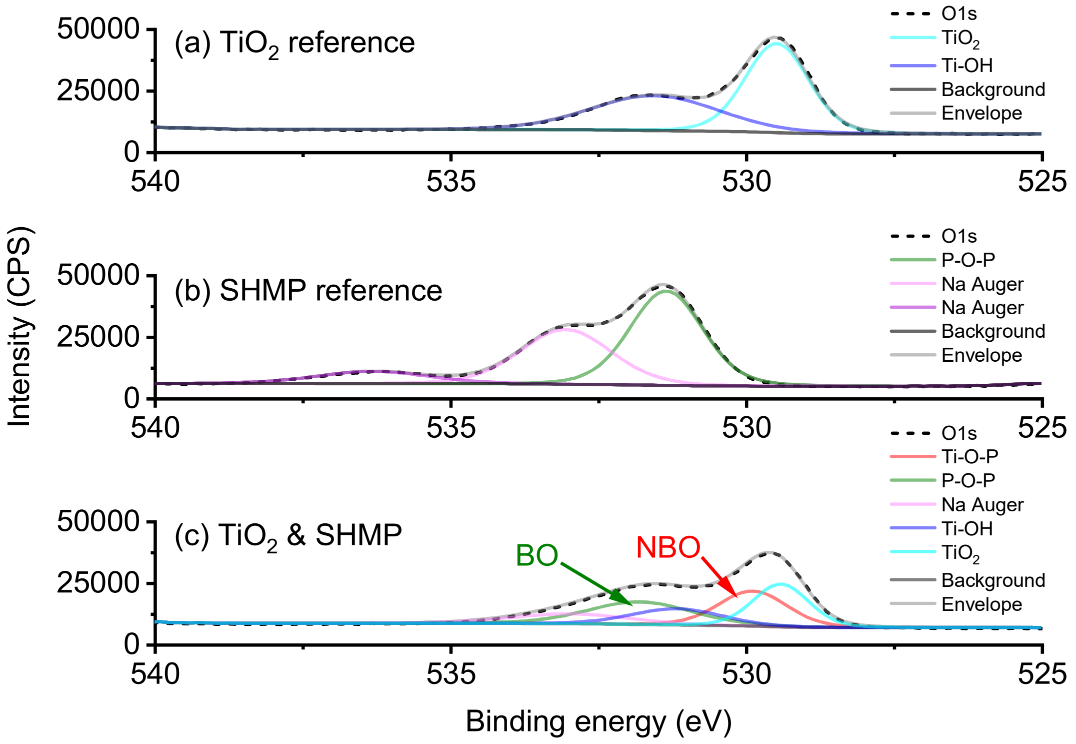

Fig. S4: O 1s peak with fitted components for (a) Al-doped TiO_2 (unmilled) reference, (b) SHMP reference and (c) Al-doped TiO_2 unmilled with adsorbed SHMP. Note: (c) is also given in the main manuscript as Fig. 6(a) and is shown here for direct comparison with reference spectra.

Further particle size data with SHMP adsorption

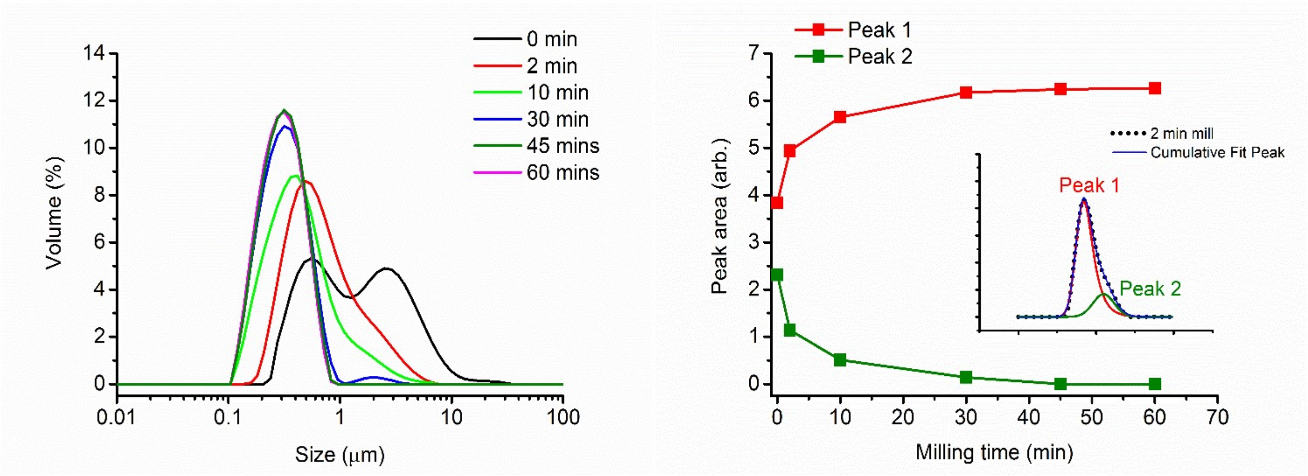

Fig. S5: Change in measured size distribution of milled Al-doped TiO_2 suspensions with adsorbed SHMP versus milling time. (a) full distributions, (b) peak deconvolution showing evolution of primary particles (Peak 1) and aggregates (Peak 2).
